# Surface Analysis of Gold Nanoparticles Functionalized with Thiol-Modified Glucose SAMs for Biosensor Applications

**DOI:** 10.3389/fchem.2016.00008

**Published:** 2016-02-29

**Authors:** Valentina Spampinato, Maria Antonietta Parracino, Rita La Spina, Francois Rossi, Giacomo Ceccone

**Affiliations:** ^1^European Commission, Joint Research Centre, Institute for Health and Consumer ProtectionIspra, Italy; ^2^Istituto di Fisica dei Plasmi, Consiglio Nazionale delle RicercheMilano, Italy; ^3^Technical Department, Nanoimmunotech S.LZaragoza, Spain

**Keywords:** gold nanoparticles, surface functionalization, XPS, ToF-SIMS, SAMs, biodetection

## Abstract

In this work, Time of Flight Secondary Ion Mass Spectrometry (ToF-SIMS), Principal Component Analysis (PCA) and X-ray Photoelectron Spectroscopy (XPS) have been used to characterize the surface chemistry of gold substrates before and after functionalization with thiol-modified glucose self-assembled monolayers and subsequent biochemical specific recognition of maltose binding protein (MBP). The results indicate that the surface functionalization is achieved both on flat and nanoparticles gold substrates thus showing the potential of the developed system as biodetection platform. Moreover, the method presented here has been found to be a sound and valid approach to characterize the surface chemistry of nanoparticles functionalized with large molecules. Both techniques were proved to be very useful tools for monitoring all the functionalization steps, including the investigation of the biological behavior of the glucose-modified particles in the presence of the maltose binding protein.

## Introduction

The characterization of the surface chemistry of nanomaterials to be used in complex environments such as biological media is a very challenging problem. In particular, in the case of nanoparticles to be employed in biosensing and medical applications the assessment of surface chemistry is of paramount importance to understand and tune interactions with complex biological environments (Grainger and Castner, 2008; Baer et al., 2010).

Amongst the different nanomaterials, gold nanoparticles (AuNPs) are the most attractive due to their extraordinary optical, electronic and molecular recognition properties, and for this reason they have been investigated as platform for many applications in various fields such as nanotechnology, materials science, life sciences and biosensors (Daniel and Astruc, 2004; Burda et al., 2005; Lee and El-Sayed, 2006; Njoki et al., 2007; Chen et al., 2008; Homola, 2008). The development of new strategies for synthesizing functionalized AuNPs is one of the most active research areas (Jain et al., 2007; Sperling et al., 2008). In fact, the ease by which the size and the shape of AuNPs can be modified by tuning the synthetic protocols makes them attractive for biosensing applications and nanotechnology in general (Niemeyer, 2001, 2003; Csaki et al., 2002; Katz et al., 2003; Parak et al., 2003). Moreover, it has been demonstrated that gold nanoparticles can be easily functionalized using different kind of ligands, polymers, biomolecules, etc. depending on the application. The strong binding affinity of AuNPs to thiols, for instance, can be exploited to assist their conjugation with biomolecules such as DNA, peptides, antibodies and proteins (Katz and Willner, 2004; Ojea-Jiménez and Puntes, 2009). Many different organosulfur compounds such as alkyl thiols and dialkyl disulphides can spontaneously form monolayers on gold substrates (self-assembled monolayers, SAMs). In fact, SAMs of alkyl thiols deposited on flat Au substrates have been extensively studied and characterized by several research groups in the last 30 years (Camillone et al., 1991; Dubois and Nuzzo, 1992; Biebuyck et al., 1994; Ulman, 1996; Schreiber, 2000).

In addition to flat and structured gold surfaces, thiol-modified nanoparticles can be also used as platforms for building molecular recognition systems by developing a hybrid nanomaterial bearing both the highly selective recognition properties of bimolecular antibodies and the unique electronic and photonic properties of nanoparticles. For instance, different kinds of nanoparticles have been applied as biomarkers and drug-delivery agents to tumors in the analysis and medical treatment of cancers (Brigger et al., 2002; Davis et al., 2008; Wang et al., 2012; Curry et al., 2014).

However, obtaining well-characterized and reproducible systems requires not only the quantification and the chemical identification of the different surface functionalities, but also the understanding of the influence of the different parameters involved in the surface chemical reactions (Grainger and Castner, 2008; Baer et al., 2010; Ambrogio et al., 2013; Neoh et al., 2015). In this respect, surface analysis spectroscopic methods such as X-ray Photoelectron Spectroscopy (XPS) and Time of Flight Secondary Ion Mass Spectrometry (ToF-SIMS) are increasingly being used to investigate the surface composition of nanomaterials ranging from metallic, oxides, semiconductors and carbon-based (Zhang et al., 2004; Okpalugo et al., 2005; Yang et al., 2005; Pinnick et al., 2008; Zorn et al., 2011; Shard, 2012; Spampinato et al., 2015). Moreover, XPS and ToF-SIMS have been used to study the interaction of biomolecules with surfaces including nanofilms and nanoparticles (Ray and Shard, 2011; Techane et al., 2011; Lebec et al., 2013; Park and Shumaker-Parry, 2014; Kim et al., 2015).

In this work, citrate stabilized AuNPs have been functionalized with a thiol-modified glucose to study the interaction with a periplasmic binding protein (Oliver et al., 2009), the maltose binding protein (MBP); this protein has a molecular weight of 48 kDa and bears the binding site in the cleft between the protein domains (Maina et al., 1988; Xavier et al., 1996; Kapust and Waugh, 1999). Thanks to its specificity and high solubility in aqueous solvents, this protein can be used as recognition element in biosensors for small analytes such as glucose (De Champdore et al., 2006; Medintz and Deschamps, 2006), for instance in monitoring sugar level in blood of patients with diabetes disease (Fonin et al., 2014).

In our study, ToF-SIMS allowed the identification of some characteristic peaks related to the coordination of thio-glucose with gold substrates as well as with the binding pocket of the protein, whilst XPS provided quantitative information about the elements content in each functionalization step. Moreover, from the XPS high-resolution spectra information about the bonds formed between the different species involved in the functionalization were also inferred.

## Materials and methods

### Sample preparation: Au NPs synthesis and functionalization

Synthesis of 15 nm sized Au NPs was carried out by modification of the procedure described by Turkevich et al. (1951), in which the gold nanoparticles were produced by the reduction of the gold salt by the sodium citrate that acts as reducing agent and stabilizer. The solution was heated up using a specialized microwave apparatus (Discover S by CEM corporation), which ensured high reproducibility, rapid and uniform heating process. In this method, 5 ml of tetrachloroauric acid trihydrate 0.01 M (HAuCl_4_·3H_2_O) (CAS No: 16961-25-4, Sigma-Aldrich, Italy) were dissolved in 95 ml of water. The solution was rapidly heated up to 97°C and held for 5 min using a maximum microwave power of 250 W under vigorous mechanical stirring. In such condition, 2.5 ml of trisodium citrate dihydrate 0.1 M (Sigma-Aldrich) were added to the solution and kept at 97°C for further 20 min. Afterwards, the solution vessel was rapidly cooled down to 40°C by a flow of compressed nitrogen.

Pristine AuNPs were used as substrates for functionalization. First, the nanoparticles were mixed overnight with a solution of 1-ß-D-thio-glucose (0.5 mM, 5n water ethanol 1:1), henceforth referred as TG, (CAS No: 10593-29-0, Mw = C_6_H_11_O_5_SNa Sigma Aldrich, Italy) so that a self-assembled monolayer could be formed on the gold surface via the S-Au bond. Then, in order to remove the excess of TG, a dialysis procedure against milliQ water was performed. The dialysis was carried out by means of cellulose membrane tubes (cut off 10000 Da, Sigma Aldrich, Italy). Before use, the dialysis tubes were washed with milliQ water and then left in boiling water for few minutes in stirring conditions. The water was poured out and the step repeated with clean water. The residual water inside the tube was removed and one end was folded and sealed with a plastic clip. 5 ml of AuNPs were then poured into the tube and the other end of the tube was also folded and clipped before being immersed in a beaker containing 250 ml of milliQ water. The sample was then allowed to dialyse for 1 h before replacing the water. The sample was subjected to total of six cycles of dialysis at which point the purified solution was analyzed and found to contain a 0.2 mM concentration of gold nanoparticles.

The TG-functionalized AuNPs were used as platform for studying the biological recognition with a protein, the maltose binding protein, henceforth MBP (AbCam, United Kingdom), which is a periplasmic binding protein involved in the transport of maltodextrins. The TG-functionalized AuNPs were mixed overnight with a MBP solution (10 mM buffer phosphate, pH7), allowing the interaction between the sugar anchored onto the surface and the protein. In order to remove the excess of protein the solution was centrifuged at 4°C and 10000 rpm for 15 min after which the majority of the supernatant solution was discarded and replaced with ultrapure water. The particles were then re-suspended by shaking before repeating the centrifugal purification step. This was repeated three times after which the suspension was then stored in the fridge at 4°C until further analysis was conducted.

### Sample preparation: Au flat substrate

For comparison with the nanoparticles, a bare gold film of about 50 nm in thickness, prepared by means of a magnetron sputtering reactor (Leybold, Germany), was used as substrate for the functionalization. First, the substrate was treated with UV-O_3_ for 30 min in order to remove possible organic contaminants on the surface, rinsed with water and ethanol and dried under a flow of high purity N_2_. The cleaned substrate was immersed overnight in a 1-ß-D-thio-glucose (TG) solution (0.5 mM, water/ethanol 1:1) to form a self-assembled monolayer of TG on the gold surface. The sample was subsequently rinsed with water and ethanol in order to remove the TG in excess and then dried under N_2_ flow. The TG-modified substrate was used as platform for biological recognition experiments with the maltose binding protein (MBP). For this, the sample was dipped overnight in a MBP solution (10 mM buffer phosphate, pH7), then rinsed first with PBS buffer, then with water and dried under N_2_ flow.

### Sample characterization

In order to assess the size distributions of the nanoparticles, Centrifugal Liquid Sedimentation (CLS) measurements were performed on the as-synthesized nanoparticle dispersions (DC24000UHR, CPS Instruments). Measurements were carried out using an 8 wt%–24 wt% sucrose density gradient with a disc speed of 22000 rpm. Each sample injection of 1 μl was preceded by a calibration step performed using certified PVC particle size standards with weight means size of 380 nm. Further characterization of the AuNPs size and morphology was performed by Scanning Electron Microscope (FEI NOVA 600I, Nanolab). The particle size distribution was also determined by dynamic light scattering (DLS, Malvern Zetasizer Nano-ZS).

ToF-SIMS analysis was conducted using a reflection-type TOFSIMS-IV spectrometer (ION-TOF GmbH, Münster, Germany) equipped with a 25 keV liquid metal ion gun (LMIG) operating with bismuth primary ions. Spectra were acquired in static mode (primary ion fluence < 10^12^ ions ·cm^−2^) in order to preserve the molecular information. During analysis, charging of the surface was prevented by applying charge compensation using low-energy (~20 eV) electron flood gun. Mass calibration of ToF-SIMS spectra was performed by using the hydrocarbon peaks CH^+^ (13 u), CH${}_{3}^{+}$ (15 u), C_2_H${}_{3}^{+}$ (27 u), C_3_H${}_{5}^{+}$ (41 u), C_5_H${}_{7}^{+}$ (67 u), and C_7_H${}_{7}^{+}$ (91 u) for positive ions spectra. Analyses were obtained from square areas of 250 × 250 μm in high mass resolution burst mode (resolution M/ΔM > 6000). Spectral interpretation was carried out using Surface Lab software v6.4 (ION-TOF GmbH, Münster, Germany). Principal Component Analysis (PCA) was performed using the NESAC/BIO MVA Toolbox (Spectragui v2.7 standalone, https://www.nb.engr.washington.edu/mvsa/nbtoolbox). Scores were plotted with the 95% confidence limit. Prior to PCA analysis, a data pre-processing step was applied to the dataset to remove variance that is not due to chemical differences between the samples. In particular, the mass spectra were first normalized to the sum of selected peaks, to account for fluctuations in secondary ion yield between different spectra and then SQRT-mean-centered.

XPS measurements were carried out with an Axis Ultra spectrometer (Kratos Analytical, Manchester, UK), using a Kα Al monochromatic source (h*v* = 1486.6 eV) operating at 150 W and X-ray spot size of 400 × 700 μm^2^ in hybrid mode. The residual pressure of the analysis chamber during the analysis was less than 8 × 10^−9^ Torr. For each sample, at least three survey spectra (0–1150 eV at pass energy of 160 eV) were acquired and used to determine the surface chemical composition (at%). In addition, one set of high-resolution spectra (analyzer pass energy at 20 eV) was recorded to obtain information about the chemical bonding of the different elements. Surface charge was compensated by a magnetic charge compensation system and the energy scale calibrated by setting the C1s hydrocarbon peak to 285 eV. The take-off angle for the acquisitions was 90° with respect to the sample surface. The acquisition time was kept below 20 min per sample to avoid possible X-ray damage and wide and core level spectra were acquired on different sample positions. The data were processed using Vision2 software (Kratos Analytical, UK) and the analysis of the XPS peaks was performed using a commercial software package (Casa XPS v2.3.16PR1, Casa Software Ltd., UK). The atomic percentages (at%) were calculated from the experimentally determined peak intensities and normalized by atomic sensitivity factors provided by Kratos Analytical. Peak fitting was performed with no preliminary smoothing. Symmetric Gaussian–Lorentzian (70% Gaussian and 30% Lorentzian) product functions were used to approximate the line shapes of the fitting components after a Shirley-type background subtraction.

## Results and discussion

The main goal of this work was to characterize and assess the surface chemistry of the different functionalization steps of Au nanoparticles with 1-ß-D-thio-glucose (TG) and the interaction with maltose binding protein (MBP). However, in order to discuss the data related to the AuNPs, we consider it useful to first present the results related to the functionalization of gold films as this will help in addressing some important issues related to the functionalized Au particles.

### Functionalization of gold flat substrates

**Figure 1:**
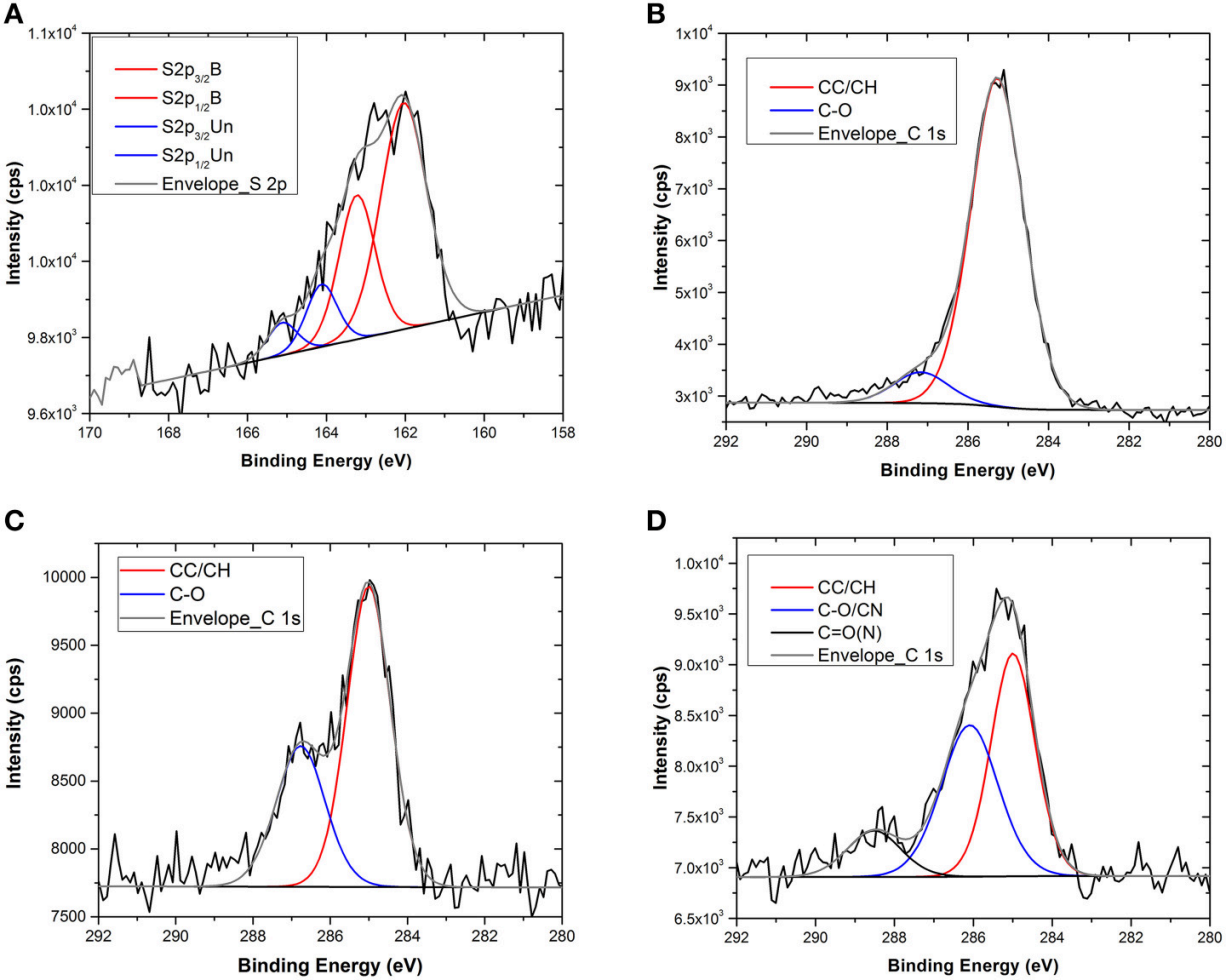
**S2p and C1s core level spectra of the flat gold substrate before and after TG functionalization (A–C) and after interaction with MBP (D)**.

XPS and ToF-SIMS measurements were performed after each functionalization step in order to obtain information about the functionalization and the chemistry of the modified surface. In Table 1 the surface compositions obtained from the XPS data are reported, whilst in Figure 1 the C1s and the S2p core level spectra recorded on the Au flat substrate, before and after the reaction with the thio-glucose, are shown.

**Table 1:** **Surface compositions of the different Au flat samples obtained from XPS analysis**.

**Samples**	**Elements line**					**S2p fitting**				**C1s fitting**		
	**O1s**	**N1s**	**C1s**	**S2p**	**Au4f**	**2p3/2 1**	**2p1/2 1**	**2p3/2 2**	**S2p1/2 2**	**C0 CC**	**C1 C-O C-N**	**C2 CON/ C = O**
	**Concentration at%**											
Au flat	1.54 (0.5)	−	34.91 (1.8)	−	63.55 (1.4)					97.61 (0.4)	2.39 (0.4)	−
Au+TG	3.36 (0.6)	−	40.38 (0.5)	2.63 (0.1)	53.65 (0.2)	54.25 (0.2)	27.12 (0.1)	12.43 (0.5)	6.21 (0.1)	86.10 (1.1)	13.91 (1.1)	−
Au+TG+MBP	5.90 (0.3)	3.93 (0.5)	42.94 (0.4)	2.60 (0.4)	44.64 (0.01)					74.14 (2.2)	14.78 (0.9)	11.08 (1.2)

(Standard Deviation in brackets).

After the TG functionalization, an increase in oxygen and carbon content, due to the TG molecule, was detected, while a decrease of gold content was observed because of the presence of the self-assembled monolayer onto the surface. Moreover, after the reaction with TG, the appearance of sulfur (about 2.6 at%) was also observed, whilst the carbon and oxygen content increases. All these results indicate the presence of TG at the Au surface.

Additional information was obtained from the analysis of the C1s and S2p core level spectra (Figure 1). In particular, looking at the high-resolution XPS spectrum of S2p (Figure 1A) it is possible to conclude that the functionalization occurred via S-Au chemical bond because of the presence of the component at 162 eV. In fact, the S2p peak can be fitted with two components, each one accounting for the spin-orbit splitting doublet S2p_1∕2_ and S2p_3∕2_ (ΔE ≅1.2 eV). The first component, centered at about 162 eV, is assigned to the bound sulfur; the second one (at about 164 eV) is related to the presence of some unbound sulfur on the surface. While the first component is related to the chemisorption of thiols, the second one underlines a certain degree of weakly bound (physisorbed) thiols that remain on the surface even if the samples were carefully rinsed with ethanol and water. The presence of this component is known in literature (Lu et al., 2000; Vericat et al., 2006) and could be due to the high density of TG molecules that form the self-assembled monolayer onto the surface. Generally, for this kind of SAM, a lateral spacer that could assist the self-assembled monolayer formation by reducing the number of molecules reacting with the substrate should be used (Cyganik et al., 2004). Indeed, since TG is a very small molecule that forms a film with thickness of less than 1 nm, the use of a spacer could provide higher flexibility and stability to the system. However, since the purpose of this study was to perform surface analysis, it was decided to reduce the system complexity by limiting as far as possible the number of functionalization steps. In any case, from the S2p fitting, it results that the percentage of S2p bound to the surface was definitely higher that the unbound one (about 80 vs. 20%) indicating the successful occurrence of the functionalization. Moreover, analysis of the C1s core level spectra before and after the reaction with the TG thiols revealed an approximately six-fold increase of the component related to the C-O bonds (286.5 eV) attributable to the presence of the TG thiols (Figures 1B,C, Table 1).

**Figure 2:**
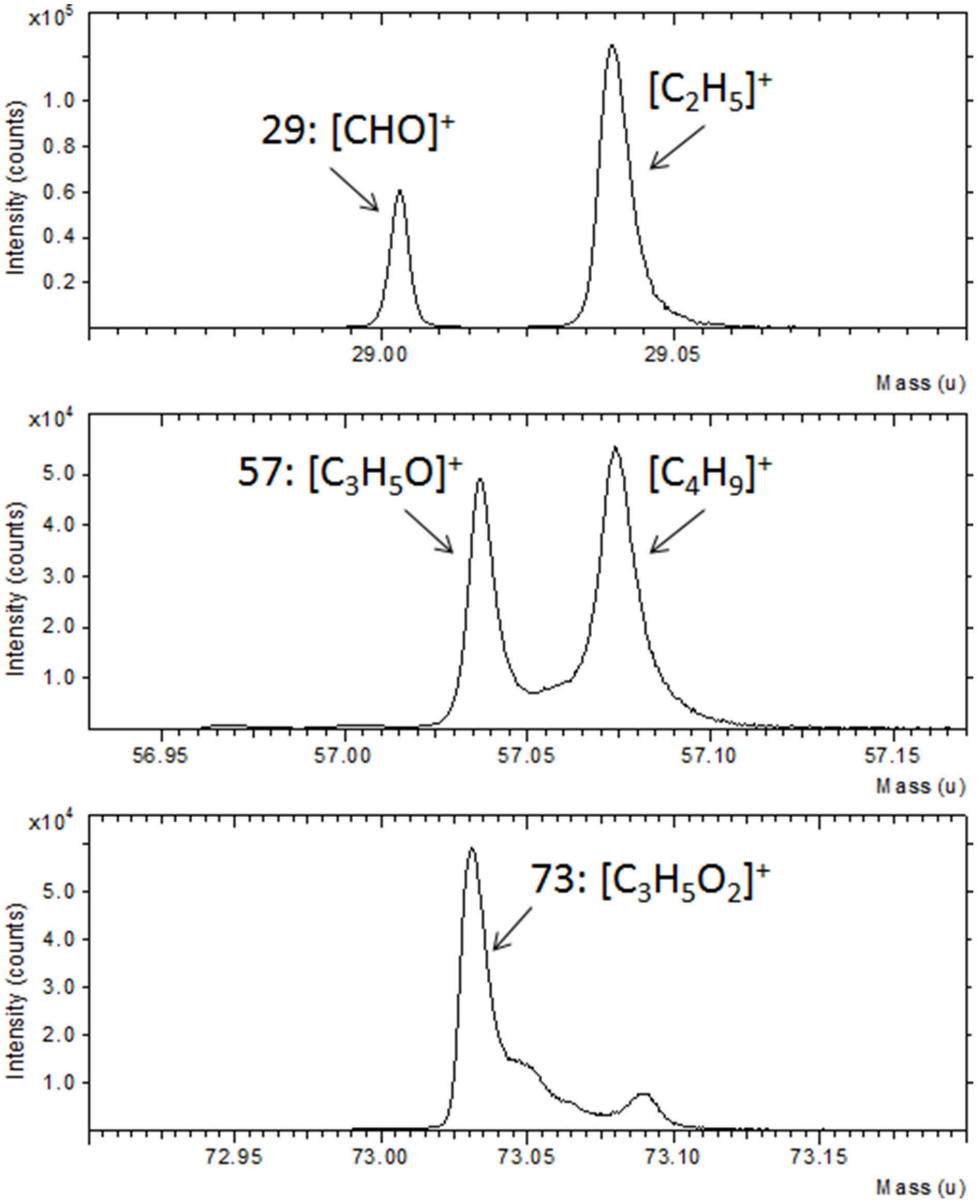
**Portion of ToF-SIMS positive spectra of Au film after reaction with TG thiols**.

Further confirmation of the TG functionalization was obtained by the ToF-SIMS analysis. In Figure 2 a portion of the ToF-SIMS positive spectrum of the TG-modified Au substrate is shown (the entire spectrum is reported in Figure SI). Some characteristic peaks, containing C, H, and O, coming from the fragmentation of the sugar, are shown. In particular, it is possible to identify the fragments [CHO]^+^ at 29 u, [CH_3_O]^+^ at 31 u, [C_2_H_5_O]^+^ at 45 u, [CH_3_O_2_]^+^ at 47 u, [CH_5_O_2_]^+^ at 49 u, [C_3_H_5_O]^+^ at 57 u, [C_2_H_4_O_2_]^+^ at 60 u, [C_2_H_5_O_2_]^+^ at 61 u, and [C_3_H_5_O_2_]^+^ at 73 u. These mass peaks were not detected during the analysis of the gold substrate suggesting the success of the TG functionalization process.

After the reaction with the MBP the surface composition change drastically as shown in Table 1. The presence of nitrogen and the increase of carbon and oxygen together with the decrease of gold signal indicate the presence of the protein on the Au surface. Moreover, the persistence of S2p signal indicates that the interaction with the MBP occurred through the TG thiol SAMs. Further, evidence of the interaction with the MBP was found in the analysis of the C1s high-resolution XPS spectra (Figure 1D). The C1s peak can be fitted with three components: C0 at about 285 eV related to the C-C/C-H hydrocarbon bonds, C1 at about 286.5 eV ascribed to the C-O and C-N bonds, and C2 at about 288.5 eV relative to the C = O and C-N-O bonds. The bare gold substrate shows the presence of the first two components and this can be explained considering the small amount of oxygen (less than 2%) detected already on the bare substrate. However, the C1 component increases (from 2 to 14%) when the functionalization with the TG occurs, because of the oxygen atoms present in the TG molecules. The third component C2 is visible only for the sample functionalized with the MBP and can be related to the peptide bond involved in the protein structure (Vanea and Simon, 2011).

**Figure 3:**
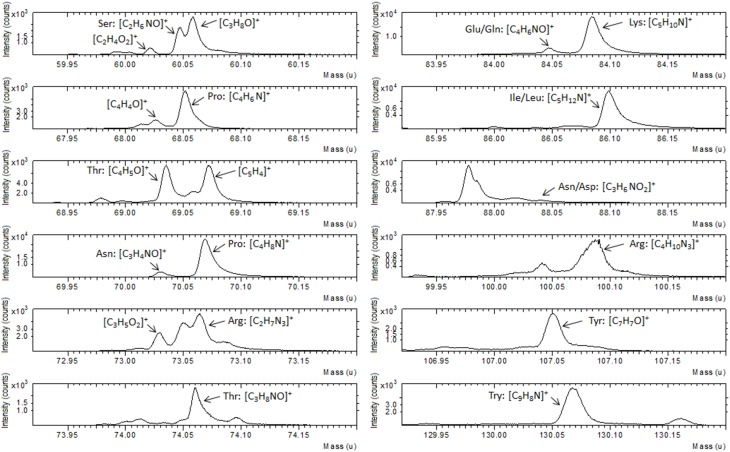
**Portion of ToF-SIMS positive recorded on Au film functionalized with TG thiols and after reaction with the MBP**.

ToF-SIMS data support the XPS result as illustrated in Figure 3, where a portion of positive spectrum in the mass range between 1 and 200 u is shown. This portion of the spectrum was analyzed for the interaction of the Au-TG surface with the MBP, since it is well known from the literature (Wagner and Castner, 2001) that protein fragmentation leads to the formation of molecular fragments related to the constituent amino acids of the protein. For example peaks at 60 u can be ascribed to serine, the ones at 68 and 70 u to proline, peaks at 69 and 74 u to threonine, peaks at 73 and 100 u can be related to arginine, peak at 82 u to lysine, peak at 88 u either to asparagine or aspartic acid and peak at 130 u to tryptophan (Wang et al., 2004).

Overall our results on the Au film indicate that the functionalization with TG thiols can be performed with a relatively simple process and that the interaction with MBP does not influence the Au-S bonding between the TG and the Au surface. These findings are a good starting point to explore the possibility of applying a similar process to gold nanoparticles.

### Functionalization of gold nanoparticles

The size and morphology of synthesized nanoparticles was characterized by means of DLS, CPS, and SEM. The results are presented in Table 2, whilst a typical CLS determined particle size distribution and SEM images are reported in Figure SI1.

**Table 2:** **Size characterization of pristine gold nanoparticles: *d*_*CLS*_ (nm) represents the average size of the AuNPs calculated by CLS, σ_*DLS*_ (nm) is the size distribution by CLS calculated at Half Height Width (HHW) of the AuNPs peak**.

	***d_*CLS*_ ± σ_*CLS*_* (nm)**	**DI*_*CLS*_* (nm)**	***d_*DLS*_ ± σ*_*DLS*_** (nm)**	***PDI_*DLS*_* (nm)**	***D_*SEM*_ ± σ*_*SEM*_** (nm)**
15 nm AuNPs	11.0 ± 1.4	1.05	14.0 ± 0.2	0.03	14 ± 2

DI_*CLS*_ (nm) is the CLS Polydispersity Index expressed as the ratio D_*w*_/D_*n*_ (where the D_*w*_ is the average particle size calculated from the particle weight-size distribution while D_*n*_ is the average particle size calculated from the equivalent number-size distribution); d_*DLS*_ (nm) is the average size calculated by DLS; s_*DLS*_ is the standard deviation between several measurement; PDI_*DLS*_ is the dimensionless polydispersity index; d_*SEM*_(nm) and s_*SEM*_ are respectively are the average size of the AuNPs calculated by SEM, and the analysis is performed by ImageJ software.

To perform the surface analysis measurements, 100 μl of suspension were spotted on titanium films (about 70 nm in thickness deposited by means of a magnetron sputtering reactor) and dried under a fume hood for 1–2 h, then inserted in the load-lock chamber (p ~ 5 × 10^−7^ Torr) where they were degassed overnight before ToF-SIMS and XPS analysis.

In Table 3 the atomic compositions of the Au nanoparticles, obtained from the XPS wide spectra, before and after the surface functionalization and interaction with MBP, (see also Figure SI2) are presented.

**Table 3:** **Surface compositions of Au nanoparticles before and after functionalization with TG and interaction with MBP**.

**Samples**	**Elements line**								**S2p fitting**			
	**Na1s**	**O1s**	**N1s**	**Ti2p**	**C1s**	**Cl2p**	**S2p**	**Au4f**	**2p3/2 1**	**2p1/2 1**	**2p3/2 2**	**S2p1/2 2**
	**Concentration at%**											
Ti substrate	−	56.93 (0.3)	−	22.31 (0.1)	20.77 (0.3)	−	−	−				
AuNPs pristine	21.83 (0.5)	36.06 (0.7)	−	2.89 (0.6)	34.93 (0.6)	2.74 (0.4)	−	1.55 (0.2)				
AuNPs+TG	11.56 (1.9)	34.99 (1.7)	−	4.86 (1.0)	41.49 (1.5)	0.31 (0.03)	0.49 (0.2)	6.30 (1.3)	47.40 (0.2)	23.69 (0.1)	19.28 (0.2)	9.64 (0.1)
AuNPs+TG+MBP	−	29.97 (0.4)	7.39 (0.5)	ND	55.88 (0.5)	−	ND	6.76 (0.8)				

(Standard Deviation in brackets).

**Figure 4:**
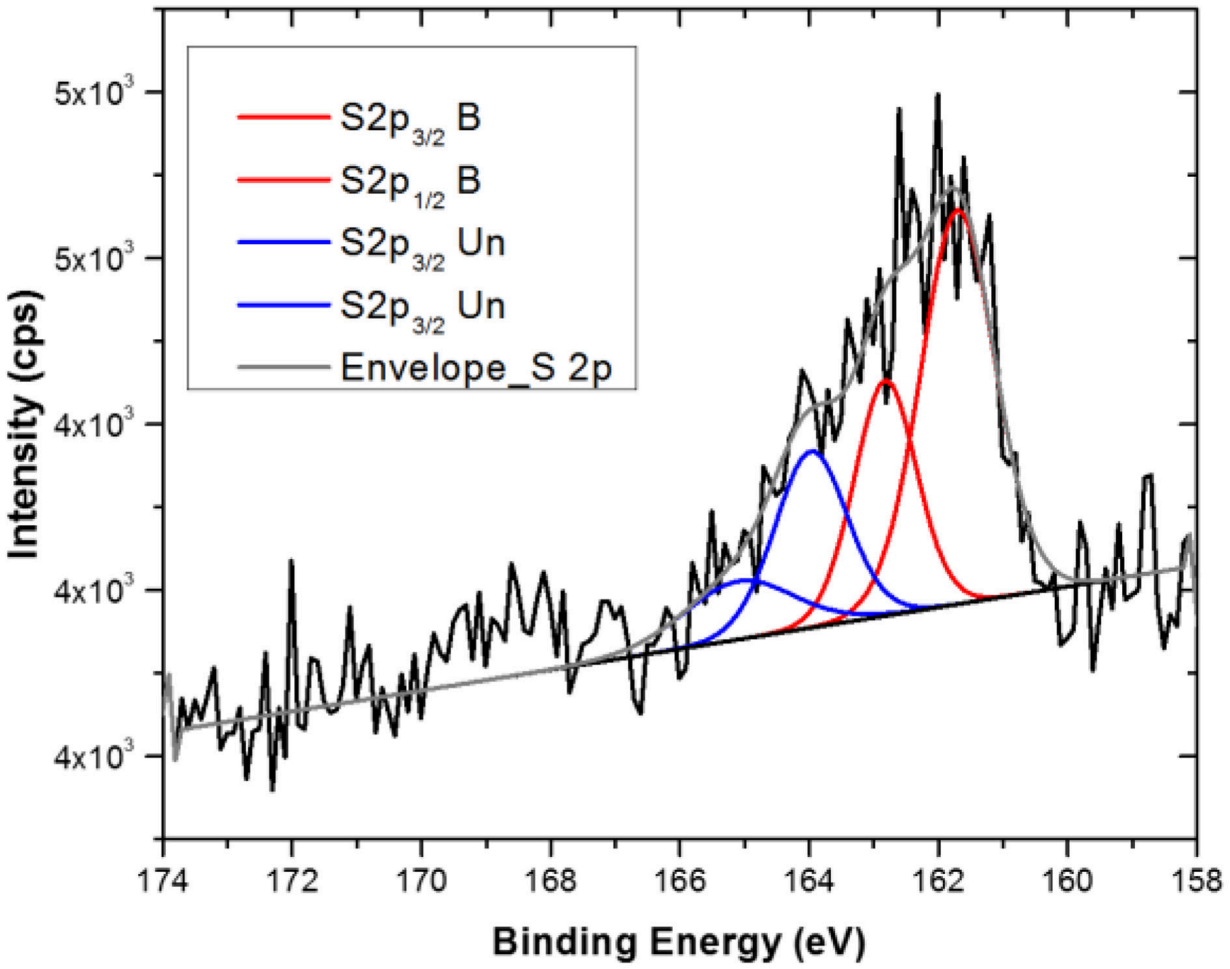
**S2p XPS Core level spectra of Au NPs after functionalization with TG thiols**.

**Figure 5:**
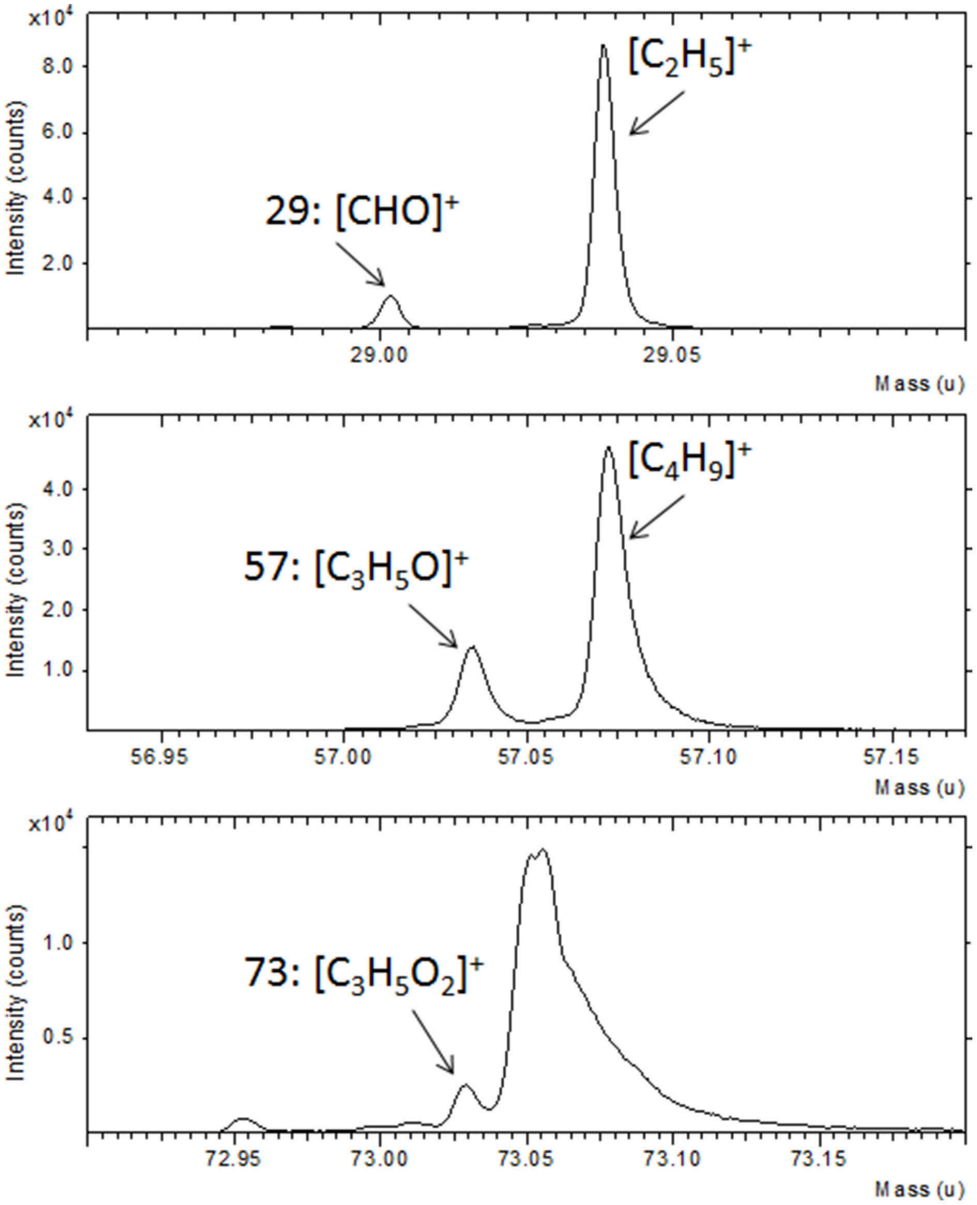
**Portions of a positive ToF-SIMS spectrum recorded on Au NPs after functionalization with TG thiols**.

As can be read from the table, the titanium substrate contains around 57 and 21% of oxygen and carbon respectively, which makes the discussion about the functionalization process based on these two elements difficult and ambiguous. Moreover, the pristine Au NPs show quite high amount of Na, C, and O that strongly mask the Au signal. This can be attributed to a quite thick layer of citrate present on the nanoparticles. Looking at the gold and sodium (as counter ion for the citrate stabilizer) percentage before and after the reaction with the TG, it is quite clear that a displacement of the citrate stabilizer by the sugar is occurring. In fact, a decrease of Na1s from 22 to 12 at% and a correspondent increase of Au4f from 2 to 6 at%, indicates a partial removal of the thick citrate layer during the functionalization process and the subsequent dialysis washing procedure. A small amount of chlorine (less than 3%) was detected on the pristine AuNPs, which is probably a residue of the HAuCl_4_ precursor used in the particles synthesis. However, after reaction with the TG the Cl2p is reduced to less than 1 at%, suggesting that this element is largely displaced during the reaction with TG thiols. As already observed in the case of the flat substrate, after the reaction with TG the appearance of S2p signal is detected at the nanoparticles surface. However, the amount of sulfur is much lower than in the case of the flat substrate indicating a lower degree of TG functionalization of the AuNPs. Despite this low sulfur amount, analysis of the S2p core level spectrum (Figure 4) provides a sound proof that TG is mostly chemisorbed onto the Au NPs surfaces. In fact, the S2p peak can be again fitted with two components (each one composed of a spin-orbit splitting doublet) as already described for the flat substrate: the first component, centered at about 162 eV, related to the bound TG thiols, whilst the second component due to the weakly bound thiols, indicating that despite the dialysis cleaning process about 30% of unbound TG thiols is still present on the Au nanoparticles surface. The fact that the Au NPs show a lower degree of functionalization could be expected because of the presence of the citrate that needs to be displaced by the TG thiols. In fact, in some recent studies Park and Shumaker-Parry have shown that the citrate is not readily displaced by thiols suggesting that a less dense functionalization occurs on citrate stabilized nanoparticles with respect to flat substrates ToF-SIMSn analysis support the XPS data as illustrated in Figure 5 where a portion of the positive ToF-SIMS spectrum of the TG modified AuNPs is reported. As can be seen the same peaks related to the TG fragmentation already observed for the flat substrate (Figure 3) are detected (Park and Shumaker-Parry, 2014, 2015).

After interaction with MBP, the composition of the Au NPs surfaces changes quite drastically (Table 3). In particular, a nitrogen content (up to 7 at%), almost double than for flat Au substrate, (Table 2) is detected. Moreover, the decrease of the Au signal and the increase of the carbon content are also observed. These data prove the presence of the MBP on the surface of the nanoparticles. In addition, the Na1s signal disappears, whilst the oxygen signal decreases, indicating a possible displacement of the remaining citrate during the interaction with the protein and the subsequent washing procedure. Since, in comparison with the case of the flat Au film the Au NPs appear to have a lower TG functionalization density, it is likely that part of the protein is interacting directly with the surface of the nanoparticles.

Additional evidence of the presence of the MBP was found in the analysis of the C1s high-resolution XPS spectra (Figure SI3), even if the presence of the citrate makes the interpretation of the spectra less straightforward. As in the case of Au flat substrate, after the reaction with the TG thiols, an increase of the component related to the C-O moieties (286.5 eV) of the thioglucose is observed, whilst the increase of the component attributable to CO(N) bonds (~288.5 eV) is detectable after the interaction with MBP.

**Figure 6:**
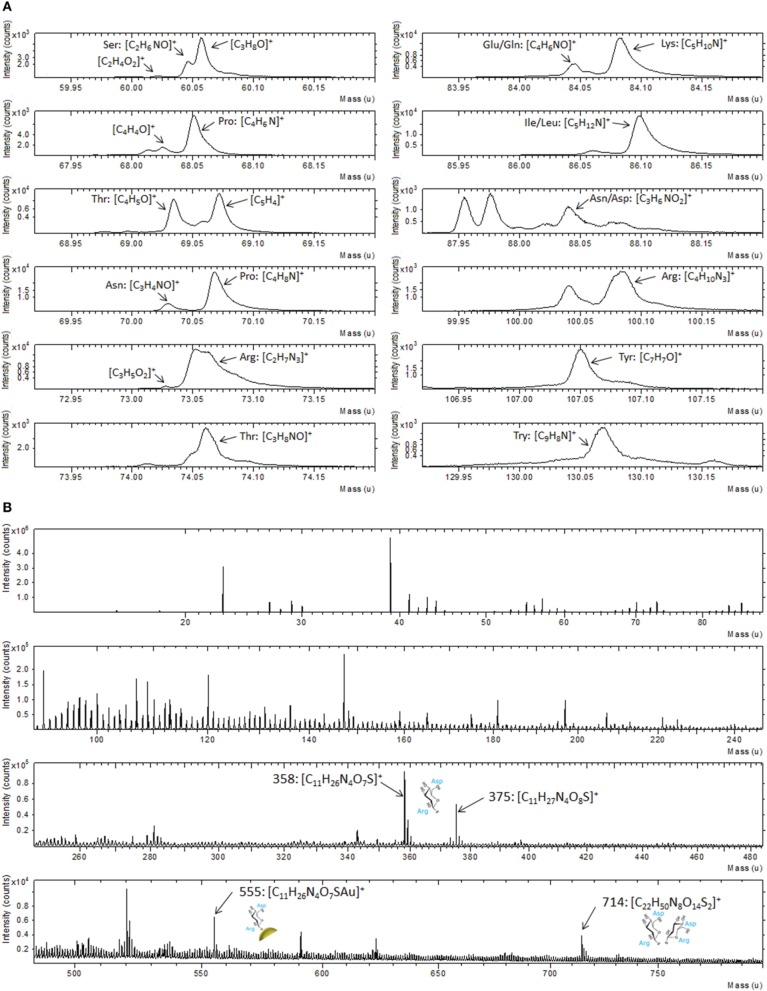
**(A)** Portions of positive ToF-SIMS spectrum recorded on Au NPs after functionalization with TG thiols and interaction with MBP; **(B)** Full positive ToF-SIMS spectrum with the high mass peaks related to the MBP-nanoparticles fragments.

Analysis by ToF-SIMS provided strong support to the XPS data also in the case of Au NPs. In particular, following the results shown above, we have concentrated our attention on the fragments related to the different amino acids in the mass range between 1 and 200 u (Figure 6A). A similar fragmentation pattern, including peaks attributable to the same amino acids already detected in the case of the flat Au film is observed (Figure 3). Moreover, the analysis of the entire spectrum, reported in Figure 6B reveals also some fragments in the mass range between 300 and 800 u. These peaks can be attributed to the TG molecule interacting with one aspartic acid and one arginine residue. In particular, the peak at 358 u corresponding to the fragment C_11_H_26_N_4_O_7_S can be ascribed to the TG molecule interacting with one aspartic acid and one arginine residue, while the same fragment bearing additional oxygen and cationized with gold is be detected at 375 and 555 u, respectively. Furthermore, at 714 u the fragment C_22_H_50_N_8_O_14_S_2_ that can be attributed to the respective dimer is also observed. These assignments take into account the geometry of interaction between the sugar and the protein. In fact, the binding pocket of the MBP exposes some specific amino acidic residues that bind the sugar, as described in literature (Herman et al., 2006; Denis Bucher et al., 2011).

### Principal component analysis of ToF-SIMS data

As reported above, some of the ToF-SIMS data in the case of AuNPs could give ambiguous information due to the presence of citrate stabilizing anions and the complexity of the spectra.

**Figure 7:**
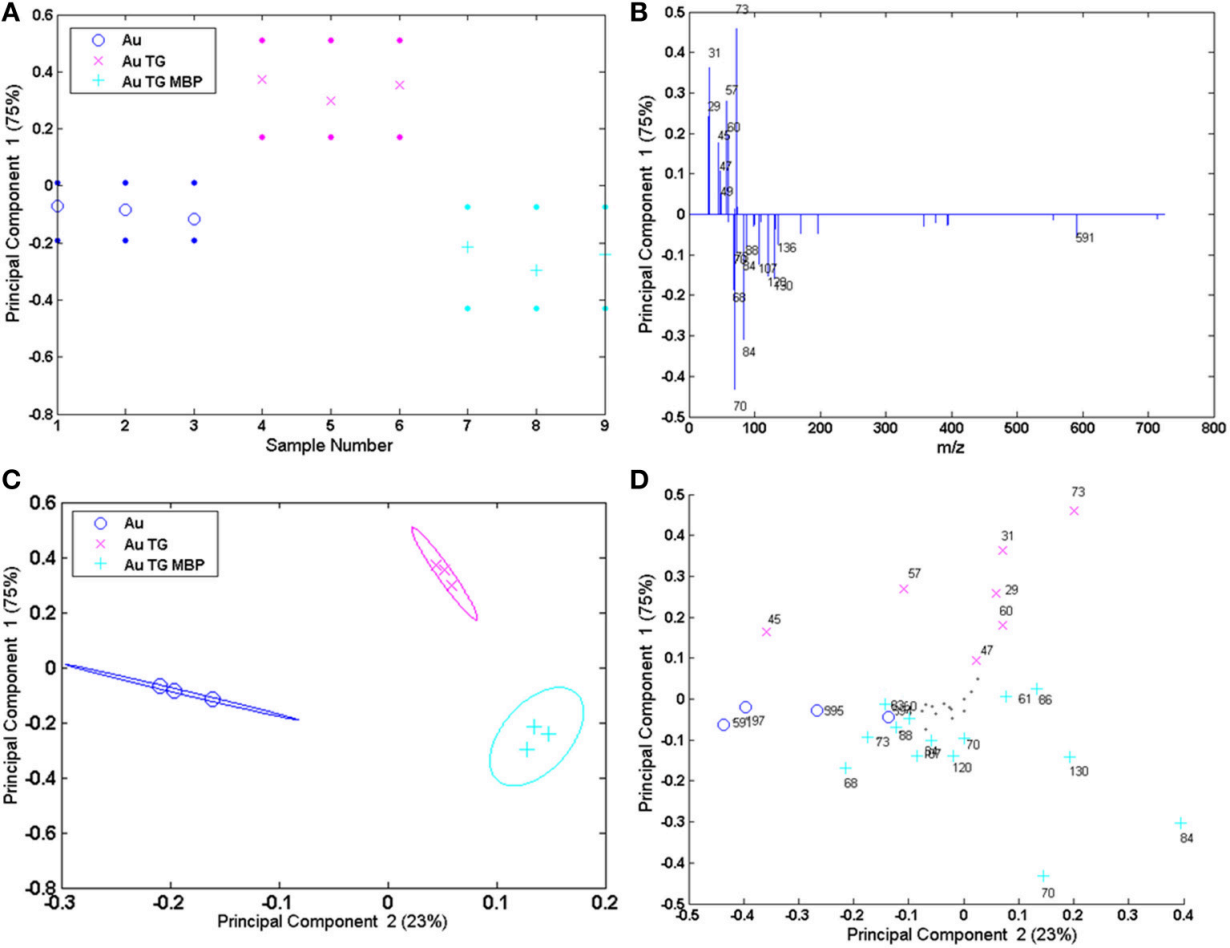
**PCA results of the Au flat samples: (A,B) PC1 scores and corresponding loading plots; (C,D) PC1 vs. PC2 scores and corresponding loading plots**.

One possible solution for extracting useful information from these complex datasets is the application of multivariate analysis (MVA) techniques and in particular the Principal Component Analysis (PCA) (Jackson, 1991; Anderson, 2013). PCA is a useful tool that can help in determining from which peaks the variability between samples arises after the surface modifications and it has been successfully applied in several studies involving ToF-SIMS analysis (Lockyer and Vickerman, 2005; Graham et al., 2006; Tyler et al., 2007; Kim et al., 2008; Yuta Yokoyama et al., 2015). In this paper PCA was carried out using the peak list reported in Table SI. In particular, 9 peaks ascribed to the TG fragmentation, 21 peaks corresponding to the amino acids fragments and 4 peaks related to the gold substrate were selected. Moreover, the four peaks ascribed to the fragments related to the interaction between the protein-binding pocket and the sugar molecules were also included in the peak list (Table SI). The PCA procedure was applied both to the spectra recorded on flat Au film and on Au NPS. However, it should be underlined that since some of the peaks ascribed to the TG can be due to the citrate fragmentation, a caution was necessary when analysing the data related to the AuNPs. Figure 7 shows the scores and the loadings plots for the first and the second principal component (PC1 and PC2) related to the flat gold sample. PCs 1 and 2 capture, respectively, 75 and 23% of the variance in the data set and are able to group the three positive ion spectra according to their different functionalization.

PC 1 differentiates the flat gold substrate and the system reacted with TG and MBP (negative scores and loadings) from the gold functionalized with the sugar (positive scores and loadings). In fact, negative loadings refer to peaks related to the amino acidic residues of the protein and gold clusters, while positive loadings refer to peaks containing only H, C, and O, thus related to the TG fragmentation. Considering the PC1 against PC2 further information can be obtained. In fact, PC2 allows separation of the bare gold substrate (PC2 negatively loaded scores and loadings) from the functionalized one (PC2 positively loaded scores and loadings).

**Figure 8:**
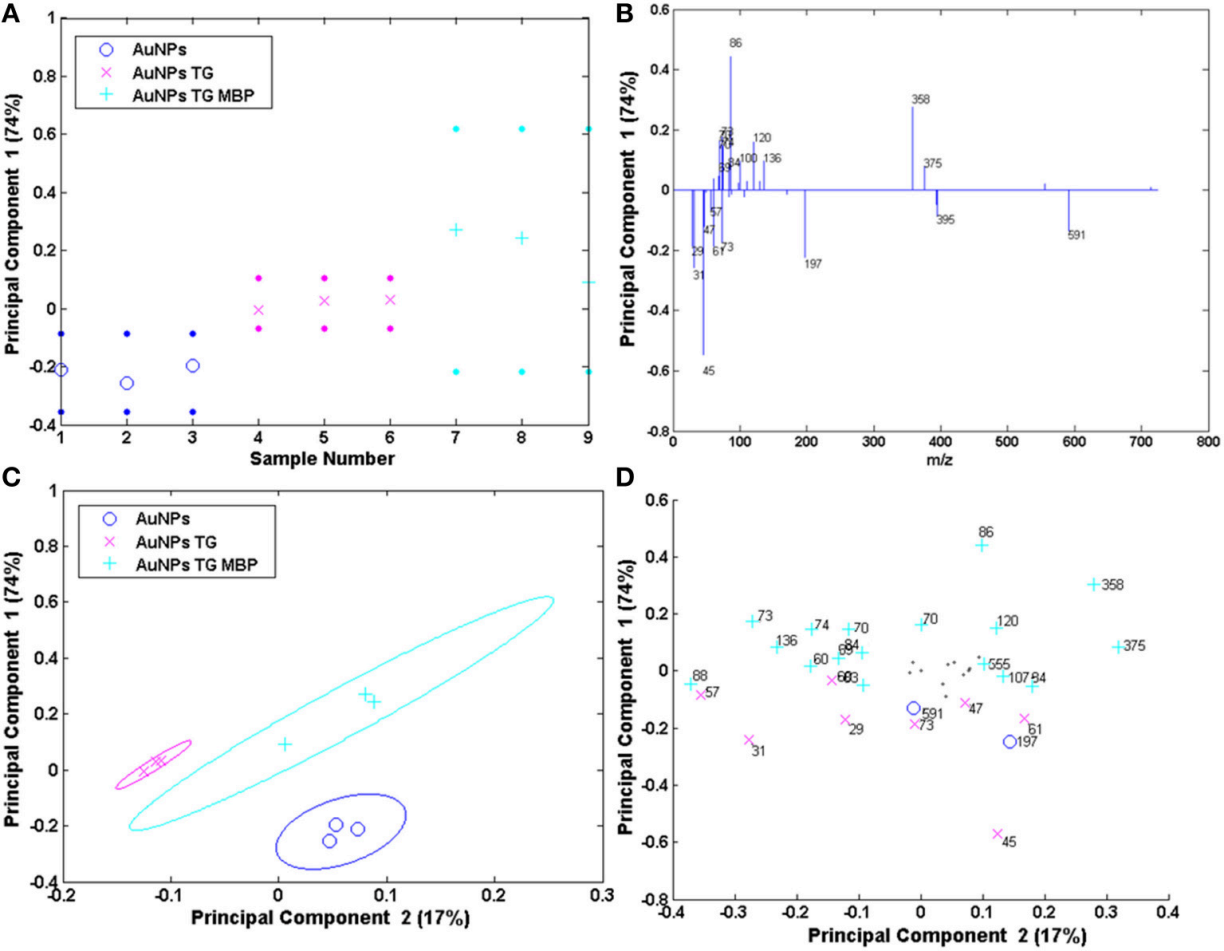
**PCA results of the Au NPs samples: (A,B) PC1 scores and corresponding loading plots; (C,D) PC1 vs. PC2 scores and corresponding loading plots**.

The results of the PCA analysis of the AuNPs are reported in Figure 8. The scores and the loadings plots for PC1 and PC2 capture 74 and 17% of the variance in the data set, respectively, and are able to group the three positive ion spectra according to their different functionalization. In particular, PC1 differentiates the AuNPs reacted with TG and MBP (positively loaded scores and loadings) from the pristine AuNPs and the ones functionalized with the sugar (scores and loadings negatively loaded). In fact, the peaks with positive loading values are related to the amino acidic residues of the protein and to the peaks related to the interaction between the TG and the MBP binding pocket, while negative loading values refer to peaks containing only H, C, O, and Au corresponding to the TG fragmentation and gold clusters. As reported above, in the case of AuNPs both TG and citrate could desorb from the surface with very similar fragmentation patterns. This could explain why these two systems lay in the portion of the plot loaded with the same polarity. However, considering PC1 against PC2 plots, it is possible to discriminate which peaks are more likely due to the citrate stabilizing agent with respect to those related to TG fragments. In fact, peaks 45, 47 and 61 u are negatively loaded in PC2 projection and are consequently more representative of the citrate stabilized AuNPs. Moreover, the peaks 358 and 375 u, related to the complex between the TG and the binding pocket of the protein, are detected in the loadings plots both for PC1 and PC2 as representative peaks of the system AuNPs-TG-MBP, thus confirming the results reported above.

The results obtained in this work indicate that the potential use of TG modified gold nanoparticles as biosensor platform for the detection of MBP could offer advantages due to their large surface area and functionalization flexibility. However, it should be underlined that each functionalization step requires carefully control to assess reproducibility and avoid variation in the MBP structure and specificity. Our results indicate that the TG is interacting with the MBP binding pocket, thus likely preserving the MBP structure and efficiency. In fact, it is known that not all the citrate is displaced during the gold nanoparticles functionalization with thiols (Park and Shumaker-Parry, 2014), leaving a proportion of the surface free to interact with other biomolecules and maybe lowering the sensing efficiency. However, if a careful optimization of the functionalization procedure is carried out more than 60% of the Au available sites could be occupied by thiols providing a good platform for biomolecule attachment (Park and Shumaker-Parry, 2015).

## Conclusions

In this paper we have reported the surface analysis of gold substrates (both as film and nanoparticles) after functionalization with 1-ß-D-thio-glucose (TG) and subsequently after interaction with maltose binding protein (MBP) solution. In particular XPS and ToF-SIMS analysis were carried out after each reaction step to assess chemical changes and to identify possible sources of contamination. The data show that the process of functionalization works quite well in the case of the Au flat surface with the possible formation of an ordered TG layer with which the MBP can interact. On the other hand, in the case of Au nanoparticles, a lower density of TG functionalities was observed, resulting in a lower interaction with MBP molecules. However, from the ToF-SIMS data it was possible to determine that the reaction of TG with MBP occurred with specific amino acid residues present in the binding pocket of the protein.

The method presented here illustrates a valid approach for surface analyses of functionalized nanoparticles and the results obtained underline the possibility of using the developed system in biodetection field.

More in general, the present work shows the importance of careful assessment of the surface chemistry of nanomaterials especially when used in complex environments such as biological media.

## Author contributions

Single authors contributed to the present papers as follows: VS carried out part of the functionalization experiments, performed all XPS and ToF-SIMS analysis and data treatment and wrote the paper; MP conceived and performed the functionalization experiments; RL did the gold nanoparticles synthesis and characterization; FR supervised the work and revised the paper; GC wrote the paper and supervised the surface analysis work.

### Conflict of interest statement

The authors declare that the research was conducted in the absence of any commercial or financial relationships that could be construed as a potential conflict of interest.
