# Supplementary material for: Surface Analysis of Gold Nanoparticles Functionalized with Thiol-Modified Glucose SAMs for Biosensor Applications
Source: Front Chem. 2016 Feb 29;4:8. doi: 10.3389/fchem.2016.00008 (PMC4770020; doi:10.3389/fchem.2016.00008)
Supplement: Supplementary file 1 [file DataSheet1.docx]

**Surface analysis of gold nanoparticles functionalized with thiol-modified glucose SAMs for biosensor applications.**

**V. Spampinato^1,2^, M. A. Parracino^1,3^, R. La Spina^1^, G. Ceccone^1*^ and F. Rossi^1^**

**^1^ European Commission, Joint Research Centre, Institute for Health and Consumer Protection, Ispra (VA), Italy**

**Supporting Information**

| **Mass (u)** | **Ions** | **Assignments** |
| --- | --- | --- |
| 29.0015 | CHO+ | TG/citrate |
| 31.0172 | CH_3O^+^ | TG/citrate |
| 45.0353 | C_2H_5O+ | TG/citrate |
| 47.0142 | CH_3O_2+ | TG/citrate |
| 49.0314 | CH_5O_2+ | TG/citrate |
| 57.0355 | C_3H_5O+ | TG/citrate |
| 60.0217 | C_2H_4O_2+ | TG/citrate |
| 60.0468 | C_2H_6NO+ | serine (Ser, S) |
| 61.0302 | C_2H_5O_2+ | TG/citrate |
| 68.0547 | C_4H_6N+ | proline (Pro, P) |
| 69.0339 | C_4H_5O+ | threonine (Thr, T) |
| 70.0322 | C_3H_4NO+ | asparagine (Asn, N) |
| 70.0708 | C_4H_8N+ | proline (Pro, P) |
| 73.0303 | C_3H_5O_2+ | TG/citrate |
| 73.0627 | C_2H_7N_3+ | arginine (Arg, R) |
| 74.0607 | C_3H_8NO+ | threonine (Thr, T) |
| 83.0499 | C_5H_7O+ | valine (Val, V) |
| 84.0467 | C_4H_6NO+ | glutamic Acid (Glu, E)/glycine (Gly, G) |
| 84.0874 | C_5H_10N+ | lysine (Lys, K) |
| 86.0997 | C_5H_12N+ | isoleucine (Ile, I)/leucine (Leu, L) |
| 88.0374 | C_3H_6NO_2+ | asparagine (Asn, N)/aspartic acid (Asp, D) |
| 98.026 | C_4H_4NO_2+ | asparagine (Asn, N) |
| 100.0824 | C_4H_10N_3+ | arginine (Arg, R) |
| 107.0523 | C_7H_7O+ | tyrosine (Tyr, Y) |
| 110.074 | C_5H_8N_3+ | histidine (His, H) |
| 120.0838 | C_8H_10N+ | phenylalanine (Phe, F) |
| 130.0676 | C_9H_8N+ | tryptophan (Trp, W) |
| 132.0539 | C_9H_8O+ | phenylalanine (Phe, F) |
| 136.0724 | C_8H_10NO+ | tyrosine (Tyr, Y) |
| 170.0687 | C_11H_8NO+ | tryptophan (Trp, W) |
| 196.9613 | Au+ | gold |
| 358.1968 | C_11H_26N_4O_7S+ | TG+Asp(88)+Arg(73)+H |
| 375.2078 | C_11H_27N_4O_8S+ | TG+Asp(88)+Arg(73)+H_2O |
| 393.9245 | Au_2+ | gold |
| 394.931 | Au_2H+ | gold |
| 555.1676 | C_11H_26N_4O_7SAu+ | TG+Asp(88)+Arg(73)+AuH |
| 590.8893 | Au_3+ | gold |
| 714.3933 | C_22H_50N_8O_14S_2+ | [TG+Asp(88)+Arg(73)]*2 |

Table SI: List of positive ToF-SIMS ions peaks used in the PCA calculations.


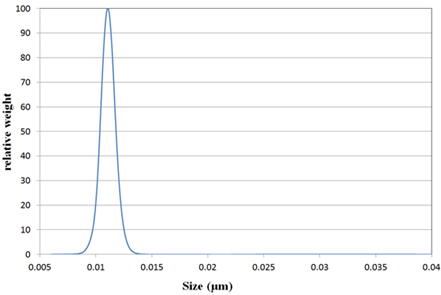

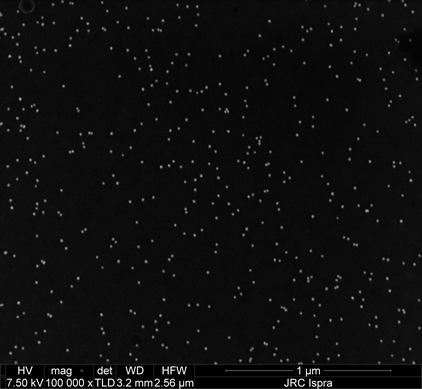


Figure SI: size characterization of the pristine gold nanoparticles: (a) CPS spectrum and (b) SEM image.


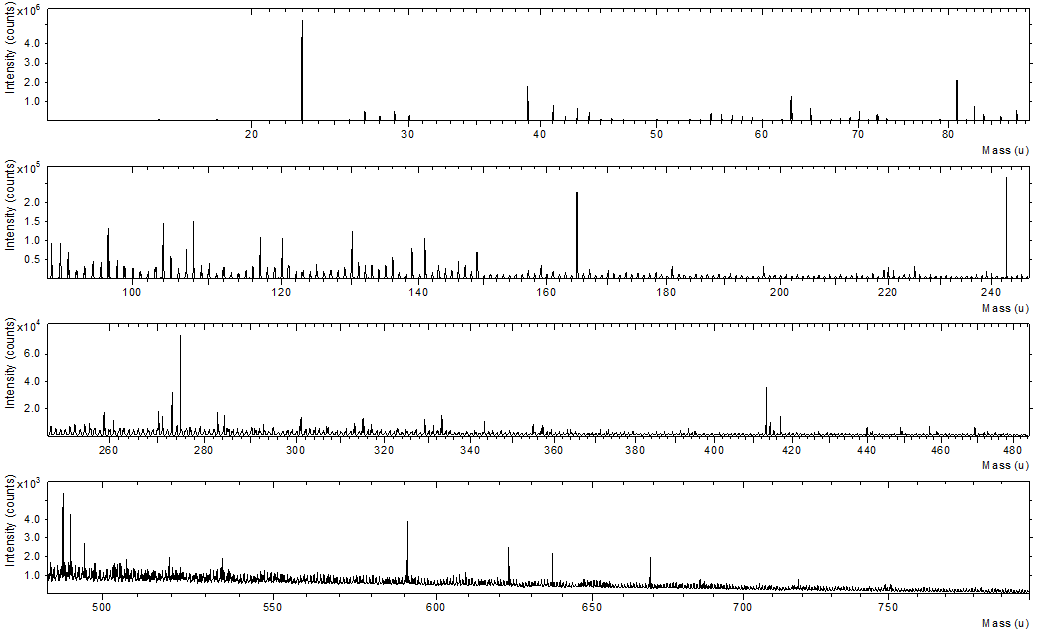


Figure SI1: Full ToF-SIMS positive spectrum of Au film after functionalization with TG thiols and reaction with MBP.


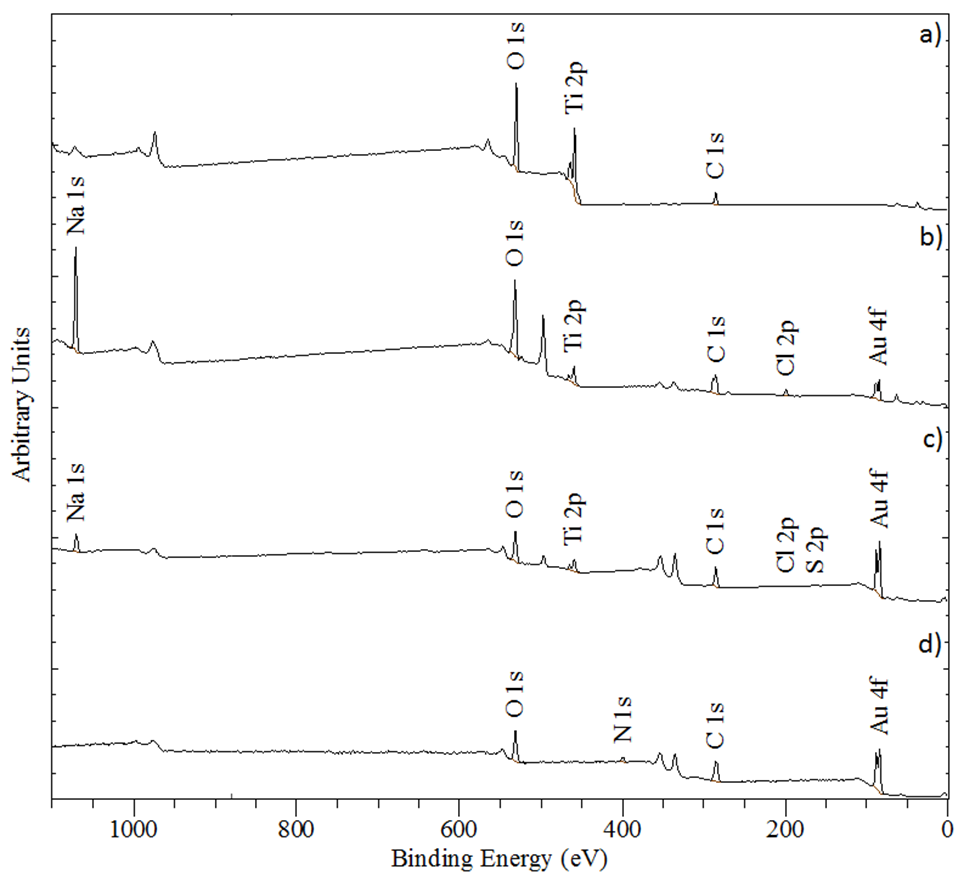


Figure SI2: XPS Survey spectra of Au Nanoparticles: (a) bare Ti Substrate; (b) Pristine Au nanoparticles; (c) after TG thiols functionalization and (d) after interaction with MBP


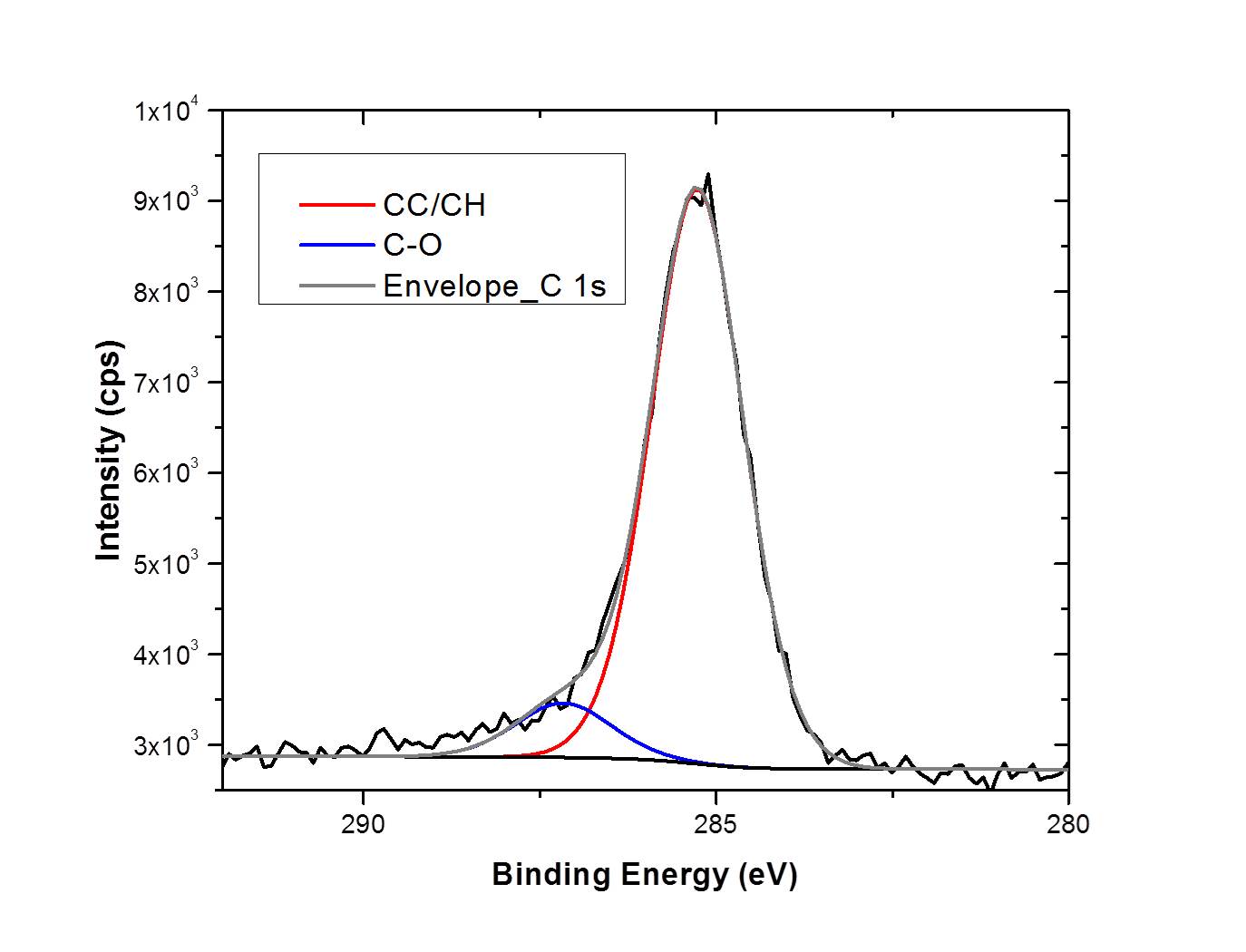

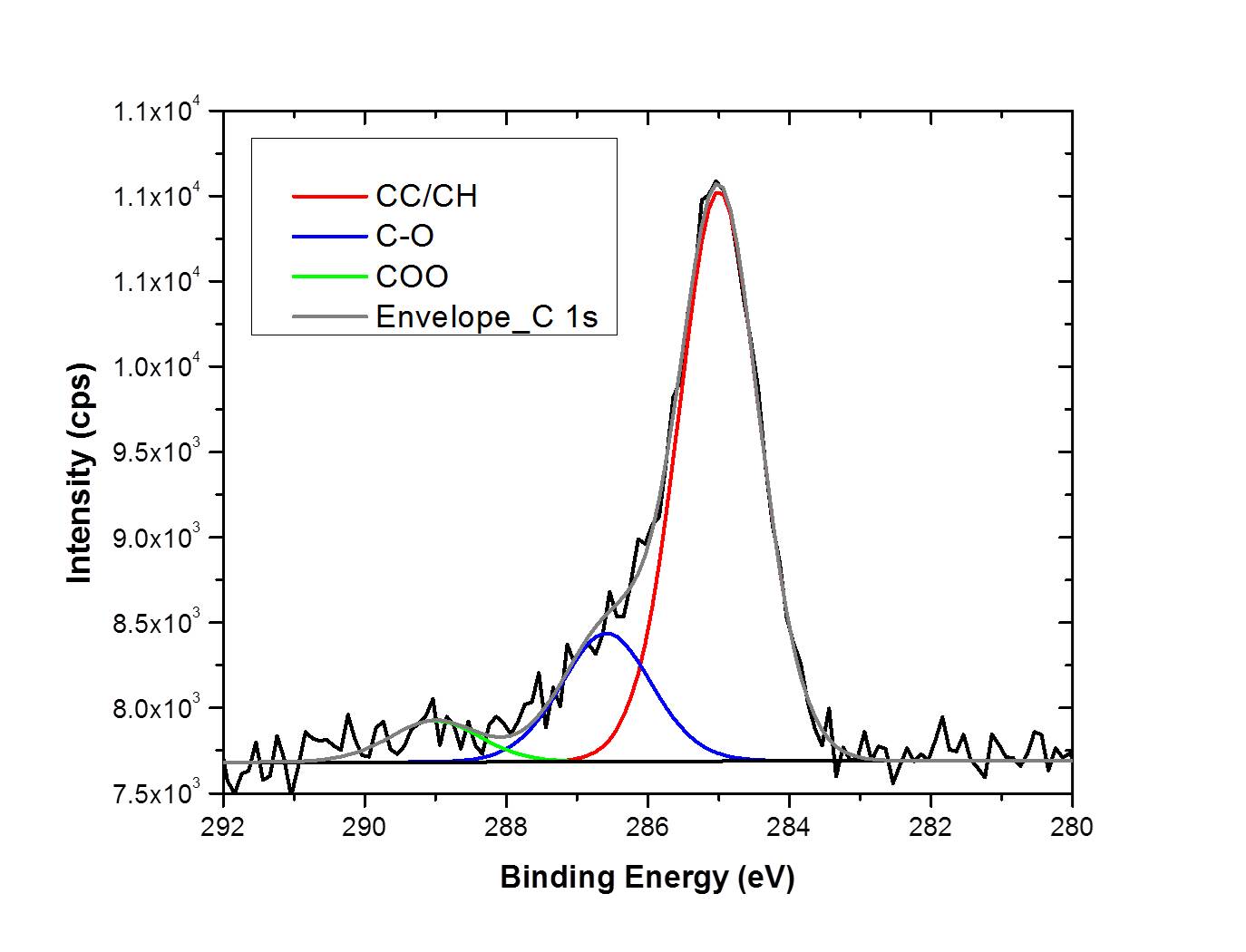

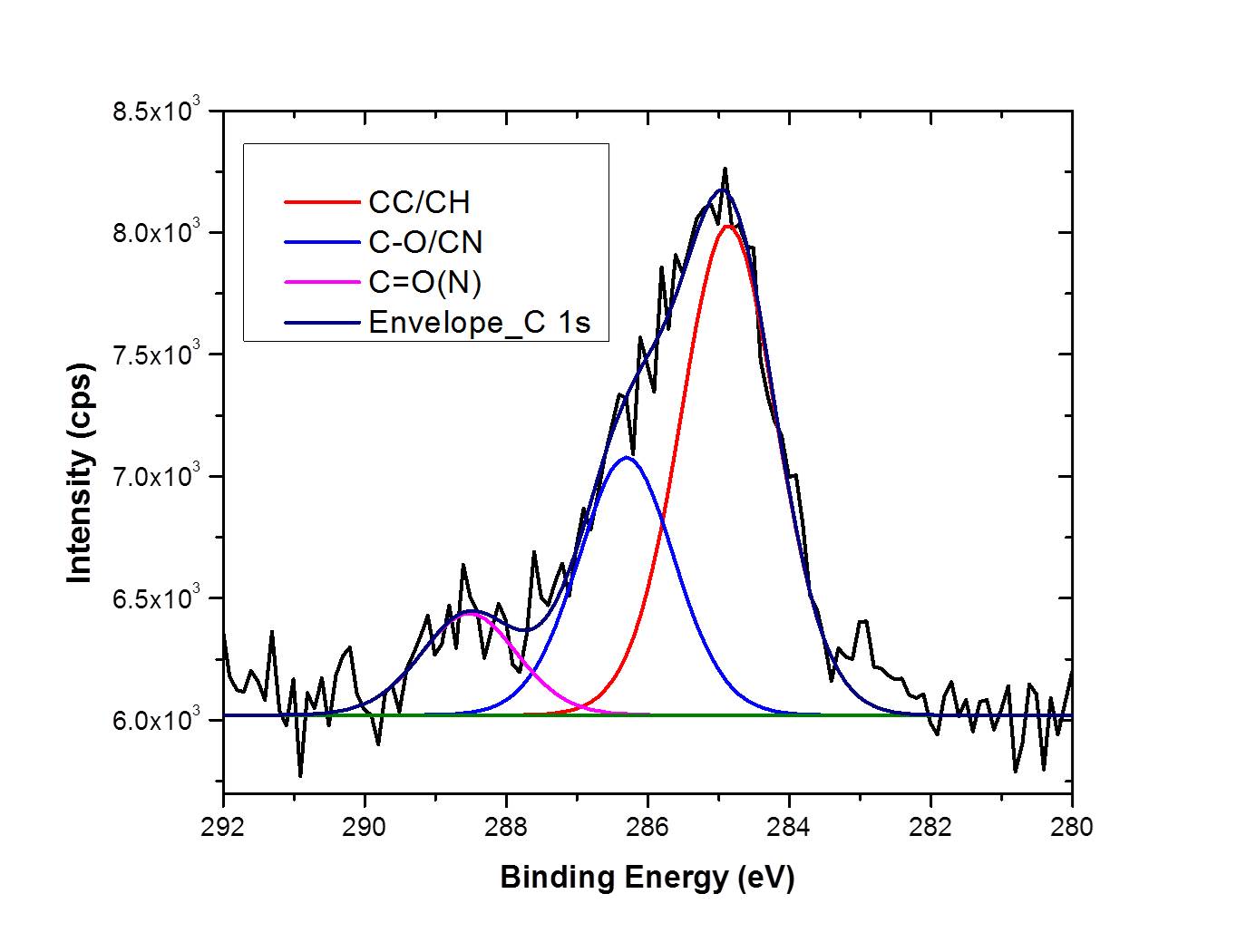


Figure SI3: C1s core level spectra of pristine AuNPs (a), AuNPs after TG functionalization (b), and TG functionalized NPs after interaction with MBP.
